# Supplementary material for: Structural congenital anomalies in resource limited setting, 2023: A systematic review and meta-analysis
Source: PLoS One. 2023 Oct 13;18(10):e0291875. doi: 10.1371/journal.pone.0291875 (PMC10575536; doi:10.1371/journal.pone.0291875)
Supplement: S2 Table — (DOCX) [file pone.0291875.s002.docx]

S2 table: NOS quality assessment of included studies on structural congenital anomalies in resource limited setting, 2023

| Studies | **Quality assessment criteria** | | | |
| --- | --- | --- | --- | --- |
|  | **Selection** | **Comparability** | **Outcome** | **Overall quality** |
| Abebe et.al ([23](#_ENREF_23)) | **** | * | ** | 7 |
| Bekalu et.al ([24](#_ENREF_24)) | **** | ** | ** | 8 |
| Eshete et.al ([25](#_ENREF_25)) | **** | * | ** | 7 |
| Feredegn et.al ([26](#_ENREF_26)) | **** | ** | ** | 8 |
| Gedamu et.al ([27](#_ENREF_27)) | **** | ** | ** | 9 |
| Jemal et.al ([28](#_ENREF_28)) | **** | ** | ** | 8 |
| Mekonen et.al ([29](#_ENREF_29)) | **** | ** | ** | 8 |
| Musa et.al ([30](#_ENREF_30)) | **** | ** | ** | 8 |
| Sileshi et.al ([31](#_ENREF_31)) | **** | * | ** | 7 |
| Taye et.al ([32](#_ENREF_32)) | **** | * | ** | 7 |

NOS: Newcastle Ottawa Scale

** Two points, *** Three points; and **** four point
