# Supplementary material for: Structural congenital anomalies in resource limited setting, 2023: A systematic review and meta-analysis
Source: PLoS One. 2023 Oct 13;18(10):e0291875. doi: 10.1371/journal.pone.0291875 (PMC10575536; doi:10.1371/journal.pone.0291875)
Supplement: S1 File — (DOCX) [file pone.0291875.s003.docx]

(((((("Congenital Abnormalities"[Mesh]) OR "Congenital Malformation"[Mesh]) OR "Congenital Anomalies"[Mesh]) AND "Risk Factors"[Mesh]) OR “associated factors” OR “predictors” OR “Determinants "[Mesh]) AND "Resource-Limited Settings"[Mesh]) OR "Developing Countries with limited resources"[Mesh]
