# Supplementary material for: Structural congenital anomalies in resource limited setting, 2023: A systematic review and meta-analysis
Source: PLoS One. 2023 Oct 13;18(10):e0291875. doi: 10.1371/journal.pone.0291875 (PMC10575536; doi:10.1371/journal.pone.0291875)
Supplement: Attachment — Submitted filename: Response to Reviewers.docx [file pone.0291875.s005.docx]

Dear editors we appreciate you considering publishing our work in one of your prestigious journal. Editors' and reviewers' issues have been thoroughly addressed, and adjustments have been made as necessary.

Dear reviewer, we are excited to incorporate your insightful criticism to improve our article. As a result, we changed the manuscript; the changes are indicated in the revised version by track changes. Below are detailed responses to the reviewer concerns.

When submitting your revision, we need you to address these additional requirements.

1. Please ensure that your manuscript meets PLOS ONE's style requirements, including those for file naming. The PLOS ONE style templates can be found at 
<https://journals.plos.org/plosone/s/file?id=wjVg/PLOSOne_formatting_sample_main_body.pdf> and 
<https://journals.plos.org/plosone/s/file?id=ba62/PLOSOne_formatting_sample_title_authors_affiliations.pdf>

**Response:** Revised was made in accordance with PLOSONE formatting samples.

2. We noticed you have some minor occurrence of overlapping text with the following previous publication(s), which needs to be addressed:

- <https://doi.org/10.1016/S0095-4543(21)00887-3>
- <https://doi.org/10.1016/j.heliyon.2022.e11657>

In your revision ensure you cite all your sources (including your own works), and quote or rephrase any duplicated text outside the methods section. Further consideration is dependent on these concerns being addressed.

**Response**: The manuscript was revised based on the advice provided and altered as necessary. YFG is the corresponding author of this document and the second study listed with doi above, and both studies' designs are meta-analyses. Even with considerable cutting and paraphrasing, this might result in some text overlap on the methods and materials section.

3. Thank you for stating the following financial disclosure:

The funders had no role in study design, data collection and analysis, decision to publish, or preparation of the manuscript.

At this time, please address the following queries:

a) Please clarify the sources of funding (financial or material support) for your study. List the grants or organizations that supported your study, including funding received from your institution. 
b) State what role the funders took in the study. If the funders had no role in your study, please state: “The funders had no role in study design, data collection and analysis, decision to publish, or preparation of the manuscript.”
c) If any authors received a salary from any of your funders, please state which authors and which funders.
d) If you did not receive any funding for this study, please state: “The authors received no specific funding for this work.”

Please include your amended statements within your cover letter; we will change the online submission form on your behalf.

**Response**: Amendments were made based on the recommendations.

4. We note that you have indicated that data from this study are available upon request. PLOS only allows data to be available upon request if there are legal or ethical restrictions on sharing data publicly. For more information on unacceptable data access restrictions, please see <http://journals.plos.org/plosone/s/data-availability#loc-unacceptable-data-access-restrictions>.

In your revised cover letter, please address the following prompts:

a) If there are ethical or legal restrictions on sharing a de-identified data set, please explain them in detail (e.g., data contain potentially sensitive information, data are owned by a third-party organization, etc.) and who has imposed them (e.g., an ethics committee). Please also provide contact information for a data access committee, ethics committee, or other institutional body to which data requests may be sent.

b) If there are no restrictions, please upload the minimal anonymized data set necessary to replicate your study findings as either Supporting Information files or to a stable, public repository and provide us with the relevant URLs, DOIs, or accession numbers. For a list of acceptable repositories, please see <http://journals.plos.org/plosone/s/data-availability#loc-recommended-repositories>.

We will update your Data Availability statement on your behalf to reflect the information you provide.

**Response**: Justification was made on the revised manuscript.

5. Your ethics statement should only appear in the Methods section of your manuscript. If your ethics statement is written in any section besides the Methods, please move it to the Methods section and delete it from any other section. Please ensure that your ethics statement is included in your manuscript, as the ethics statement entered into the online submission form will not be published alongside your manuscript.

**Response**: Corrected

6. Please include captions for your Supporting Information files at the end of your manuscript, and update any in-text citations to match accordingly. Please see our Supporting Information guidelines for more information: <http://journals.plos.org/plosone/s/supporting-information>.

**Response**: Captions for supporting files are included in the revised version of the manuscript

Reviewer #1: The authors tried to review the title which is an important public health problems. The neural tube defect is common problems in low and middle income countries due to different factors. The underutilization of health services including preconception service in this regions makes things complicated. Having the overall understanding of the problem is very relevant. However, authors should incorporate or answer the following comments, suggestions, and questions and improve the language of the manuscript to improve it, otherwise it is suitable for publication with current shape.
Title: Structural congenital anomalies in resource limited setting, 2023: A systematic review and meta-analysis
Is resource limited setting is well known in World Bank economic classification of countries? I.e., high income countries, middle income countries, low income countries etc.

**Response**: Your concern was considered significant, and the operational definition of a resource-limited setting was added to the methods and materials section in the amended paper.
Abstract
Your background claim that there is a paucity of studies with a comprehensive review of structural anomalies; hence what is this gaps has to do with pooling the existing evidences?
Make your aim in line with your title.

**Response**: The error has been fixed in the current version. It was meant to imply that there are no studies that include a comprehensive review.
Who did searching, data extraction and what type study quality check method was employed in your case?

**Response**: The initials of authors conducted searching and data extraction were described in quality assessment and data extraction subsection of the Methods and materials section. The basic quality of included research articles was evaluated using the Newcastle-Ottawa Scale and included as a supporting file in the current version.
How taking folic acid tablet during pregnancy OR =6.01 (95%CI 2.87-14.89) was positively associated with outcome in contrast to what already known?

**Response**: This study claims that **never** using folic acid was positively associated with outcome.
The authors should indicate how outcomes variables and explanatory variables such as maternal illness, birth weight, chewing chat, chemical exposure, taking folic acid during pregnancy, unidentified drugs etc. were measured.

**Response**: Those variables are described in operational definition subsection of methods and materials section.
Did your study/review support preconception care and vaccination care that you recommended?

**Response**: Vaccination was removed from the recommendation section. All statistically significant factors in this study that could result in congenital abnormalities could be avoided if the client received preconception care. That’s why preconception care included in the recommendation.

Background
Indicate the location of the evidences and its context, i.e., whether it is in the globe, LMICs, SSA etc. and also indicate type of evidence whether it is review, report, article etc.

**Response**: The background was changed to reflect this.
“……, there is no study that serves as a standard for such settings.” So are you going to conduct primary study?

**Response**: Last paragraph of the background was intensively revised.
Authors failed to indicate why they are performing this systematic review and meta-analysis. Why large geographic coverage was considered despite huge heterogeneity that it could result in?

**Response**: Even though congenital abnormalities could be significantly reduced with preconception care, providing preconception care has been hampered in all resources-limited settings which make them homogeneous. 
How authors pooling observational studies identified risk factors?

**Response**: The term "**possible** risk factors" was chosen for this study because, despite the fact that the primary studies were observational studies, it more accurately characterizes the components listed here than the term "associated factors."
Authors should have indicated how stakeholders benefitted from systematic review and meta-analysis, whether researchers, policy or program designers, clinicians, etc.

**Response**: It’s indicated in the final paragraph of the background section.
Method
Information source did not indicate the MeSH terms correctly, for instance the MeSH term for congenital abnormalities is ‘Congenital Abnormalities’ not “Congenital Abnormalities/abnormalities".
The sample search of PubMed is not well organized. The key terms are not exhaustive.
The eligibility is not well explained point by points. For instance how you managed studies that reported proportion of outcome only, non-structural congenital anomalies, etc.

**Response**: Revised and provided as a supporting file.

Where is PICO or other?

**Response**: Included in table 1
Where are study selection and data extraction subtitles?

**Response**: It’s included in quality assessment and data extraction subsection
“Egger test was done and verified that there was no small-study effects.” Don’t you think this is part of result?

**Response**: Yes it’s, and corrected accordingly.
Result
Figure 1 describe those excluded by setting and outcomes in your ‘study characteristics’ part and indicate the difference clearly. Similarly, indicate studies which were removed due to quality if any.
Table 1 indicated all studies were performed in Ethiopia, hence why you talking about resource limited setting? Why not you say simple ‘Ethiopia’. The title mislead readers.

**Response**: Studies carried out in settings with limited resources were thoroughly reviewed. Unfortunately, no studies have been conducted in environments other than Ethiopia that meet the inclusion criteria. Until articles are available, those initial research carried out in Ethiopia were thought to nominate the poor resource situation of low-income countries. Additionally, it is believed to be employed as an evidence-based practice in low resource settings to intervene with congenital abnormalities.
Discussion
The first and second paragraph is not related to discussion. The author should have focused on the summary finding in the first paragraph.

**Response**: Corrected accordingly
“Selection and characterization of included studies” what selection have to do in the result section?

**Response**: Title of the subsection was corrected.
“Possible risk factors of Congenital anomalies in low resource setting” how authors identified risk factors using cross-sectional studies?

**Response**: The goal was to use "possible risk factors" rather than "risk factors," which might be used in the same way that we say "associated factors".
Authors should link their finding to previous studies and provide the possible reason behind for the complement and disagreement of the findings. For instance how maternal illness related to tubal defect and what is the reason behind? How chat chewing related to tubal defect? Have you included studies from eastern part of the country where chat chewing is relatively high? What is the pathophysiology behind? Why <https://www.ncbi.nlm.nih.gov/pmc/articles/PMC7672758/> not included? Which specific maternal illness is associated with tubal defects? How? Why?

**Response**: We apologize for the error in omitting such a significant paper from the study. However, we have now cited it. Since the primary studies that were included stated "maternal illness during pregnancy" as a variable, without identifying the type of illness; because of this we simply generalized it and assumed that any illness should be monitored cautiously to avoid congenital abnormalities. We advocate experimental research because the primary papers pooled in this analysis were quantitative observational studies that were unable to address some of the concerns outlined above, particularly the "how" ones.
What are the implications of your findings?
Recommendations are not usually listed as you did, instead summarize in one or two sentences based on your conclusion.

**Response**: the recommendation part was revised.
Additional files
Where is searching techniques for all databases?
Where is quality assessment grading using Newcastle-Ottawa Scale?

**Response**: provided as a supporting files.
